# Nanotechnology assisted photo- and sonodynamic therapy for overcoming drug resistance

**DOI:** 10.20892/j.issn.2095-3941.2020.0328

**Published:** 2021-06-15

**Authors:** Rui Li, Zhimin Chen, Zhifei Dai, Yingjie Yu

**Affiliations:** 1 College of Life Science and Technology, Beijing University of Chemical Technology, Beijing 100029, China; 2 Department of Biomedical Engineering, College of Engineering, Peking University, Beijing 100871, China; 3 Institute of Translational Medicine, The First Affiliated Hospital of Shenzhen University, Shenzhen Second People’s Hospital, Shenzhen 518039, China

**Keywords:** Drug resistance, photodynamic therapy, sonodynamic therapy, chemotherapy, nanotechnology

## Abstract

Drug resistance is considered the most important reason for the clinical failure of cancer chemotherapy. Circumventing drug resistance and improving the efficacy of anticancer agents remains a major challenge. Over the past several decades, photodynamic therapy (PDT) and sonodynamic therapy (SDT) have attracted substantial attention for their efficacy in cancer treatment, and have been combined with chemotherapy to overcome drug resistance. However, simultaneously delivering sensitizers and chemotherapy drugs to same tumor cell remains challenging, thus greatly limiting this combinational therapy. The rapid development of nanotechnology provides a new approach to solve this problem. Nano-based drug delivery systems can not only improve the targeted delivery of agents but also co-deliver multiple drug components in single nanoparticles to achieve optimal synergistic effects. In this review, we briefly summarize the mechanisms of drug resistance, discuss the advantages and disadvantages of PDT and SDT in reversing drug resistance, and describe state-of-the-art research using nano-mediated PDT and SDT to solve these refractory problems. This review also highlights the clinical translational potential for this combinational therapy.

## Introduction

Chemotherapy, a mainstream cancer treatment, plays an important role in tackling cancer^1^. More than 200 anticancer drugs have been used clinically^2^. These drugs usually work well at early stages of disease, but more than 90% of patients show drug resistance after relapse^3^. Even patients treated with immunotherapy almost inevitably develop drug resistance in relatively short periods of time^4,5^. Because of the low therapeutic indexes of most chemotherapeutic drugs, even slight changes in the sensitivity of tumor cells can result in drug resistance. All these factors make drug resistance a major obstacle in cancer treatment^6^.

Over the past half-century, progress has been made in understanding drug resistance, thereby facilitating the development of new therapeutic strategies for overcoming this obstacle^7^. Scientists have proposed 3 major hypotheses underlying drug resistance: (1) pharmacokinetics, in which up-regulating the expression of efflux membrane proteins and detoxification enzymes leads to insufficient accumulation of drugs in tumor regions^8^; (2) tumor specificity^9^, in which genetic mutations in cancer cells are the biological basis of drug resistance: after application of chemical drugs, the tumor cells gradually acquire genetic mutations and epigenetic changes, and the elimination of sensitive subtypes leads to the development of drug-resistant tumors; and (3) the tumor micro-environment (TME)^10^, which regulates the drug sensitivity of tumor cells and promotes the development of drug resistant phenotypes^11^.

PDT, an invasive treatment for clinical cancer, has been used to reverse chemoresistance^12,13^. The 3 elements of PDT include a photosensitizer (PS), light, and oxygen. Light-activated PS transfers energy to oxygen and generates cytotoxic reactive oxygen species (ROS)^14^, which decrease the expression of membrane efflux proteins and anti-apoptotic proteins^15^. Because of the high tissue penetration of near-infrared light (NIR), various NIR-excited PSs have been developed^16^. In addition, the unique mechanism of PDT can enhance tumor sensitivity, vascular permeability, and immune responses^17,18^.

SDT is an emerging therapy, which generates ROS through a combination of low intensity ultrasound (US) (∼1 MHz) and sensitizing drugs^19^. The main advantage of SDT is that US has deep penetration in mammalian tissue (above 10 cm)^20^, thus making SDT a promising therapy for deep tumors^21–23^. Microbubbles, which have been approved as contrast agents for US diagnosis, are used to load and release oxygen under US and regulate the TME^24–27^. Because drug-resistant cells have a higher clearance rate of ROS than sensitive cells, they are more susceptible to ROS^28,29^. Hence, combining PDT or SDT with conventional chemotherapy endows conventional chemotherapy with more versatility, thereby providing an effective and facile means of overcoming drug resistance.

**Figure 1:**
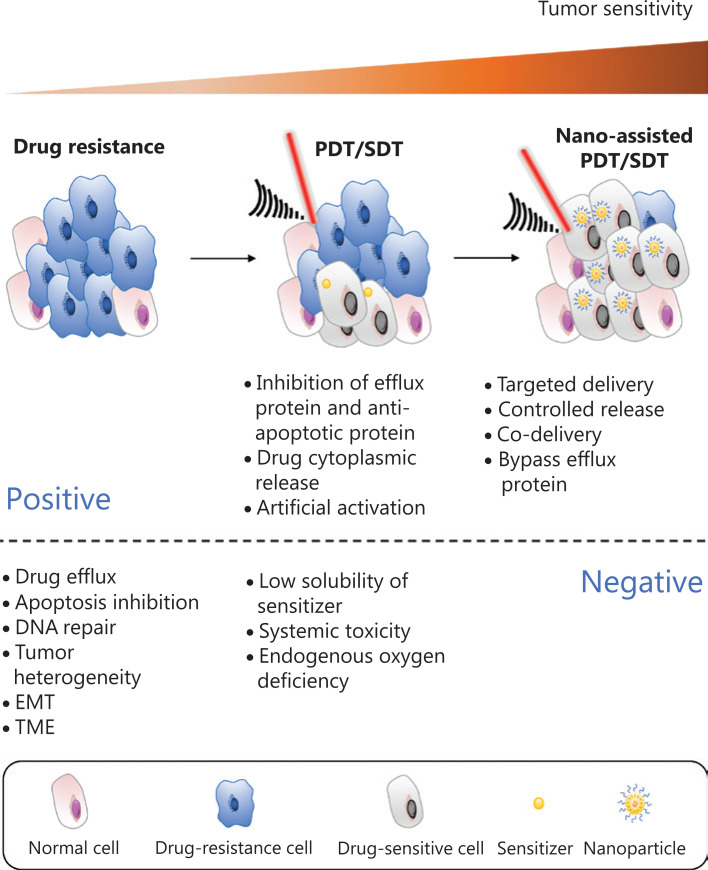
Schematic illustration of nanotechnology assisted photo- and sonodynamic therapy for overcoming drug resistance. The drug resistance of cancer cells is closely associated with drug efflux, apoptosis inhibition, DNA repair, tumor heterogeneity, tumor epithelial-mesenchymal transition (EMT), and the tumor microenvironment (TME). The application of PDT and SDT improves the sensitivity of tumors by inhibiting drug resistance-related proteins, thus artificially activating and promoting drug internalization. Nanotechnology is applied not only to bypass efflux proteins but also to facilitate targeted delivery and the controlled release of sensitizers.

Nanotechnology is the manufacturing of materials at atomic and molecular scales. Because of their unique properties, nanomaterials have been the basis for development of numerous drug delivery systems. Although drug-resistant cells are more susceptible to ROS, PDT and SDT still have several limitations that compromise their efficacy^30,31^. With the development of ideal sensitizers for PDT and SDT, light and US not only sensitize tumor cells but also trigger the release of sensitizers into the cytoplasm, thus bypassing the efflux membrane proteins and inhibiting the escape pathway and significantly enhancing drug accumulation in tumor regions^32^. The strategies of nanotechnology assisted PDT and SDT to overcome drug resistance are summarized in **Figure 1**.

## Drug resistance remains a major hindrance in cancer therapy

**Figure 2:**
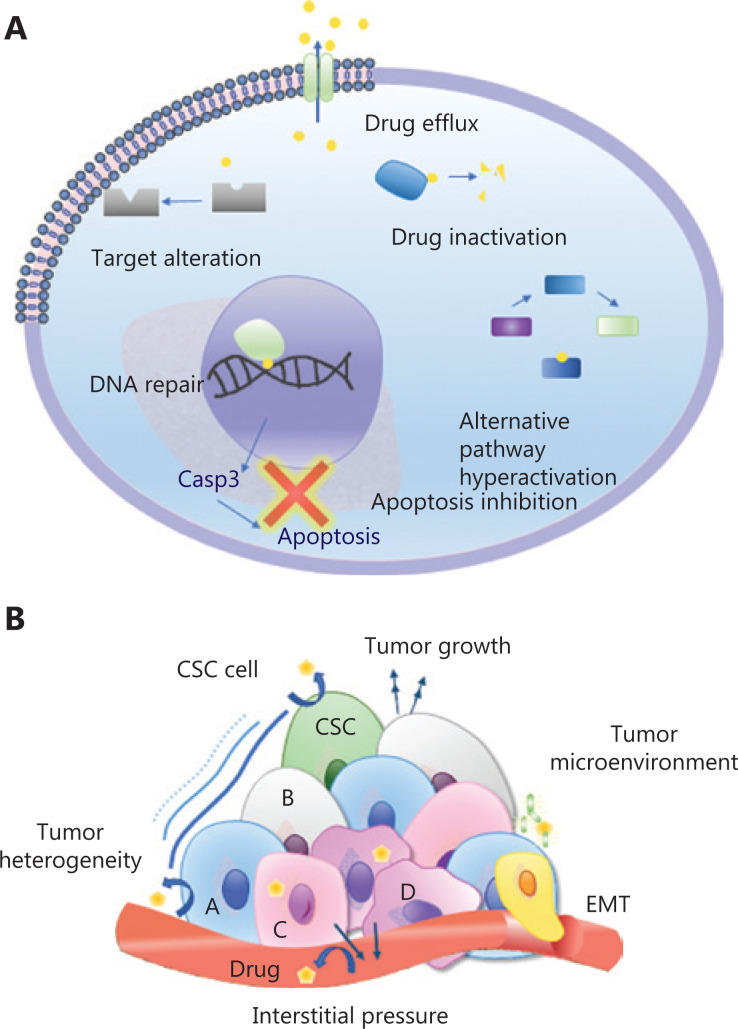
Schematic illustration of the mechanism of cancer drug resistance. (A) Common cell-intrinsic resistance mechanisms. (B) Tumor microenvironment and systemic mechanisms of drug resistance.

Because drug resistance is a major predictor of patient mortality^33,34^, understanding the mechanisms of drug resistance is crucial^35^. Resistance to a wide range of anticancer drugs is attributed to the expression of energy-dependent transporters, which eliminate anticancer drugs from cells. These transporters are called ATP binding cassette (ABC) proteins, which include multidrug resistance protein 1 (MDR-1), multidrug resistance related protein 1 (MRP-1), and ATP-binding cassette subfamily G member 2 (ABCG-2)^36^. However, other resistance mechanisms, such as insensitivity to drug-induced apoptosis, DNA repair, target alteration, alternative pathway hyperactivation, and induction of drug-detoxification, are likely to lead to anticancer drug resistance (**Figure 2A**)^37,38^.

Beyond intracellular signals, the TME and systemic factors also affect the development of drug resistance (**Figure 2B**)^39^. For example, a hypoxic environment activates hypoxia inducible factor-1 (HIF-1), which regulates the expression of MDR-1. Moreover, epithelial-mesenchymal transition (EMT) cells have similar cellular characteristics to those of cancer stem cells (CSC)^40,41^. The EMT cells decrease the efficacy of chemotherapy by releasing cytokines^42^. Importantly, tumors are extremely heterogeneous, and this aspect considerably contributes to primary or acquired resistance^43^. In a further challenge, some of these resistance pathways may result in multidrug resistance. Improved understanding of the diverse mechanisms of cancer drug resistance would aid in designing various anti-cancer therapeutic strategies to circumvent drug resistance.

## Photo- and sonodynamic therapy to overcome drug resistance

### The unique mechanisms of action of PDT in overcoming drug resistance

**Figure 3:**
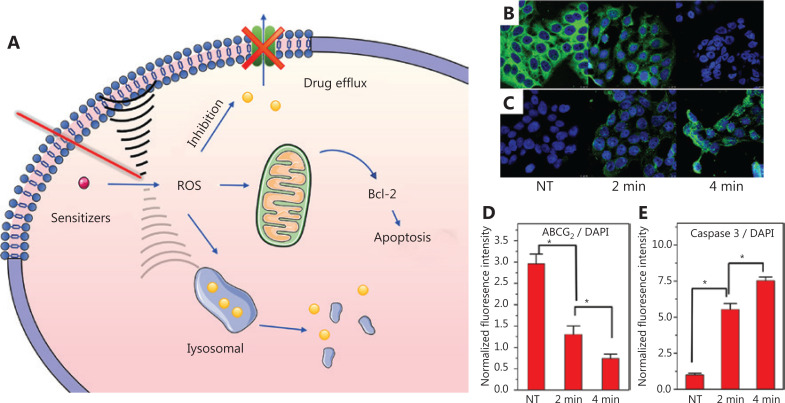
(A) Schematic diagram of PDT overcoming drug resistance at the molecular level. PDT caused decreased expression of ABCG_2_ protein (green) (B) and increased expression of Caspase-3 (green) (C) in HT-29 cells. The nucleus is stained blue by DAPI. Quantification of the ABCG_2_ (D) and caspase-3 (E) fluorescence intensity per DAPI area after PDT irradiation^50^. *Significant difference (*P* < 0.05). Copyright 2018, American Chemical Society.

PDT has attracted great attention as a promising therapy for drug-resistant tumors, because of its unique mechanisms^44^. ROS produced by PDT disrupt the original cytokine balance, transforming the tumor cells from a resistant to a sensitive phenotype (**Figure 3A**)^45^. The most effective PSs tend to be lipophilic aromatic ring systems, which are preferentially located on extranuclear organelle membranes^46,47^. Among them, the PSs located in mitochondria disrupt the membrane structure, thereby leading to a sharp decline in the levels of intracellular 5′-adenosine triphosphate (ATP) and anti-apoptotic protein Bcl-2 family proteins^48,49^. The activity of ATP-dependent ABC proteins is consequently decreased, and efflux of chemotherapy drugs is inhibited (**Figure 3B–3E**)^50–53^. Moreover, lysosomal-PDT selectively destroys the lysosomal membrane, thus bypassing protective autophagy and promoting cytoplasmic drug release^54^. In terms of the TME, vascular injury is observed after PDT, including enhanced vessel permeability and leakage, thereby improving the therapeutic index of chemotherapeutic drugs^55,56^. In addition, PDT stimulates tumors to form an inflammatory environment and induces T cells to infiltrate tumors^57^.

In summary, the unique mechanism of PDT includes: (1) reversal of chemo-resistance and sensitization of tumors to molecular inhibitors; (2) modulation of vascular permeability for enhanced drug delivery; and (3) stimulation of anti-tumor immunity. Thus, nanotechnologies to integrate the special features of PDT are promising in clinical treatment of drug resistant cancer.

### SDT reversal of drug resistance

Although SDT remains in its infancy, it has nonetheless received tremendous attention in cancer treatment. Similarly to PDT, SDT decreases mitochondrial membrane potential and oxidative phosphorylation, thus down-regulating the expression of ABC proteins^58–60^. However, the biological effects of SDT differ from those of PDT, because of the mechanical effects of ultrasonic treatment (cavitation, alternating pressure, shear stress, and acoustic current), which themselves can decrease drug resistance even without cytostatics. In drug-resistant cells over-expressing P-gp, the fluidity of the drug-resistant cell membrane decreases, thus increasing sensitivity to US^61^. Moreover, the introduction of microbubbles allows macromolecules to enter cells *via* stable cavitation^62,63^. Hence, better understanding of the ROS production mechanism in SDT would aid in the design of more effective sensitizing drugs to overcome drug resistance.

### Limitations of PDT and SDT

PDT and SDT have a strong ability to sensitize tumor cells to chemotherapy, but they nonetheless have many drawbacks, such as sensitizer aggregation, selective enrichment, and endogenous oxygen deficiency. First, the π-π aggregation of the hydrophobic sensitizer results in poor solubility in aqueous solution, thus decreasing the degree of internalization and the quantum yield of singlet oxygen^64^. Second, the different physiological distributions of sensitizers and chemo-drugs may cause systemic toxicity. Designing an appropriate system to selectively deliver sensitizing agents is urgently needed. Third, oxygen is essential for the development of drug resistance and ROS. However, hypoxia, a characteristic of the TME, not only promotes the proliferation of drug-resistant tumors but also decreases the efficiency of PDT and SDT^65^. The introduction of nanotechnology provides a new strategy to address these issues.

## Nanotechnology approaches enhance the efficacy of PDT/SDT in overcoming drug resistance

### Targeted delivery of sensitizers

**Figure 4:**
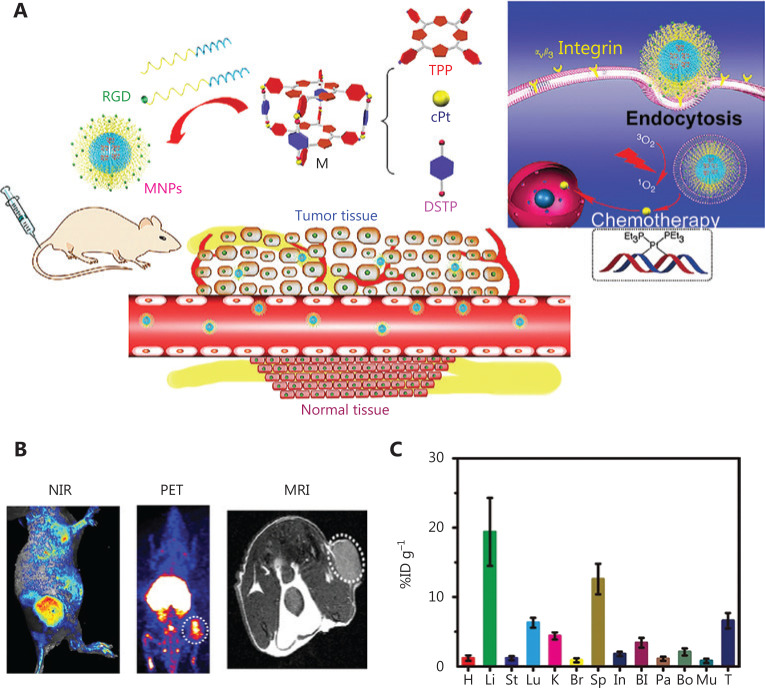
(A) Schematic illustration of nanoparticle structure and tumor site accumulation. Nanoparticles are targeted to tumors through active RGD targeting. (B) *In vivo* three-peak imaging. The white circle denotes the tumor site. (C) Imaging of U87MG tumor-bearing mice after MNP injection. The enrichment of nanoparticles in tumors can be clearly observed in (B) and (C)^91^. Copyright 2018, Nature.

To overcome the drawbacks of PDT and SDT, nanoparticles (NPs) have been used as drug delivery systems to increase the permeability, stability^66^, and solubility of sensitizers^67^ and avoid excessive drug removal^68^. Because of the lack of lymphatic drainage, tumor tissues, compared with normal tissues, tend to retain more NPs that escape from underdeveloped tumor blood capillaries. This phenomenon is termed the enhanced permeability and retention (EPR) effect^69–71^. Many studies (**Table 1**) have found that the EPR effect significantly increases drug levels in the tumor region, thereby preventing drug resistance. In sonodynamic photodynamic therapy, a new type of combination therapy^82–85^, the application of NPs has also shown excellent performance in improving drug efficacy^80,81^. Additionally, nanomedicines enter cells through endocytosis, a process independent of the MDR protein-mediated pathway^86^. After PDT (660 nm, 10 mW/cm^2^, 5 min) treatment, NPs escape from lysosomes successfully, thereby suggesting that cells absorb NPs through endocytosis and evade ABC-mediated drug resistance^87^. In comparison to the EPR effect, active targeting offers better selectivity^88–90^. The targeted cyclic peptide RGD associates with the surfaces of the NPs. As shown in **Figure 4B**, drug enrichment at tumor sites is clearly observed after injection^91^. Excitingly, the cell mortality rate has been found to significantly increase to 95.6% (61.3% for apoptosis and 34.3% for necrosis) after 671 nm light irradiation 0.1 W/cm^2^ for 3 min. These results indicate that nano-design can increase the intracellular content of sensitizers and drugs, and may serve as a convenient method for overcoming drug resistance.

**Table 1:** Nanosystems to overcome drug resistance through the EPR effect

Nanoparticles	Treatment	Sensitizers	Operating parameters	Therapeutic outcomes	References
Core–shell–shell nanoparticles (UCNPs)	PDT	RB	808 nm, 6 W/cm^2^, 5 min	PDT and chemotherapy effectively kill A2780*cis* cells (IC_50_ value 9.3-fold lower than that with cisplatin).	^72^
Graphene oxide	PDT	Ce6	470 nm, 25 mW, 5 min	NPs loaded with camptothecin and Ce6 are more easily absorbed by cells and significantly improve the anti-cancer efficacy.	^73^
Singlet-oxygen producible polymeric micelles	PDT	Ce6	670 nm, 6 mW/cm^2^, 100 s	Singlet oxygen generated by PS mediates cell membrane damage and enhances the accumulation of DOX in drug-resistant cells. In drug-resistant cells, the IC_50_ of NPs is 160 times lower than that of free DOX.	^74^
Multifunctional composite of MoS2@Fe3O-ICG/Pt(IV)	PDT/PTT	ICG	808 nm, 1 W/cm^2^, 5 min	Nanoparticles show good MR/IR/PA bioimaging effects, thus indicating that NPs can be enriched at tumor sites. The percentage ratio of apoptotic or necrotic cells can reach 86.4%.	^75^
Photosensitizer H_2_TPPS and DOX self-assembled nanoparticles	PDT	H_2_TPPS	376 nm, 40 mW/cm^2^, 10 min	The resistance of MCF-7/ADR cells to DOX is effectively reversed. The IC_50_ value is 1.49 μg/mL.	^76^
Organoplatinum (II) metallacage coated octaethylporphine (OEP)	PDT	OEP	635 nm, 0.2 W/cm^2^, 5 min	The tumor suppression rate of A2780*cis* tumor-bearing nude mice s 66.8%, a value higher than that for cisplatin (14.1%).	^77^
TiO_2_ based hydrogenated hollow nano-sound sensitizer integrating precious metal Pt and doxorubicin (HPT–DOX)	SDT	TiO_2_	1 MHz, 50% duty cycle, 1.5 W/cm^2^, 5 min	HPT-DOX generates ROS independently of endogenous oxygen and increases drug delivery to overcome chemotherapy resistance.	^78^
Pluronic F68 nanomicelles co-loaded with doxorubicin (HPDF-DOX)	SDT	HP	1 MHz, 1.5 W/cm^2^, 30 s	HPDF nanomicelles, as compared with free DOX, reverse the drug resistance of MCF-7/ADR cells, with a reversal index as high as 19.0.	^79^
5-ALA/TiO_2_ nanoparticles	SDT/PDT	TiO_2_/5-ALA	SDT: 1 MHz, 70 W, 10 minPDT: 635 nm, 150 mW/cm^2^, 1000 s	Tumor tissue is irradiated with lasers and sonication, thus resulting in a decrease in tumor volume by approximately 50%.	^80^
Peptide amphiphile-ICG nanomicelles (PAIN)	SDT-PDT	ICG	SDT: 1 MHz, 2.4 W/cm^2^, 5 minPDT: 808 nm, 1.5 W/cm^2^, 3 min	After treatment of MDA cells with PDT and SDT, the production of ROS is almost twice that with free PDT.	^81^

### NIR/US activation release

**Figure 5:**
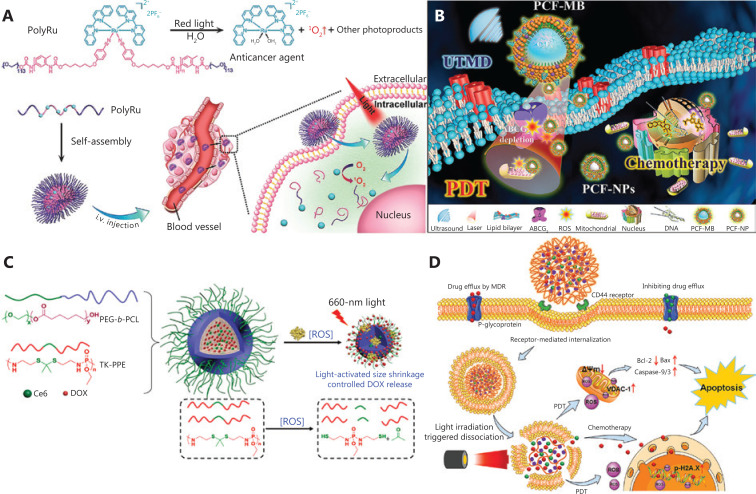
(A) The light activation process of PolyRu^98^. Copyright 2016, Wiley. (B) Ultrasound-mediated microbubble blasting and the nano-particle interaction mechanism^50^. Copyright 2018, American Chemical Society. (C) Schematic diagram of *in situ* TK bond fracture caused by ROS^103^. Copyright 2018, American Chemical Society. (D) ROS-responsive nanoplatform to promote lysosomal drug release^105^. Copyright 2018, American Chemical Society.

Nanotechnology-enabled drug delivery systems can provide spatial and temporal control for drug release. Various controlled release systems based on the TME^92^, NIR^93,94^ and US^95,96^ have been developed^97^. The NIR response has been confirmed to simultaneously activate PDT and NP disintegration at target sites. Sun et al.^98^ have synthesized a photoactivated nano-metal prodrug, PolyRu (**Figure 5A**). PolyRu can be cleaved by NIR to achieve on-demand administration, thus increasing the intracellular concentrations of drugs. PolyRu with red light irradiation (656 nm, 50 mW/cm^2^, 30 min), as compared with control treatment, has been found to decrease tumor volumes by approximately 55%. As mentioned above, the US response is more conducive to treatment of deep drug-resistant tumors, particularly with the assistance of microbubbles. The collapse of microbubbles due to sonodynamics increases vascular permeability^99^. Using this feature, Chen et al.^50^ have constructed porphyrin/camptothecin-fluorouridine triad microbubble (PCF-MB) to treat drug-resistant for breast cancer (**Figure 5B**). Ultrasound triggers the conversion of PCF-MBs into PCF-NPs, which induce greater internalization and uptake. Shi et al.^100^ have designed “US-detonated nano bombs” containing DOX, which lead to lysosomal escape and mitochondrial targeting. DOX is released from the nanobombs after US treatment (1 W/cm^2^, 120 s, at 4 h after incubation), and large amounts of DOX are observed in the nucleus.

Polyethylene glycol (PEG), which is widely used to modify nanomaterials, decreases the uptake of drugs by non-specific cells and prolongs the blood circulation time. The light-controlled shedding of PEG at desired sites has shown advantages in on-demand drug delivery based on nanocarriers^101^. The ROS-activatable thioketa (TK) bond has been used in the construction of PEG light-controlled shedding nanosystems^102^. Cao et al.^103^ have explored the polymer nanocarrier TK-PPE@ NP Ce6/DOX. Under excitation at 660 nm NIR, ROS generated by encapsulated Ce6 cleave the TK linker *in situ*, thus achieving drug remote control release through shrinking in size from 154 ± 4 nm to 72 ± 3 nm (**Figure 5C**). Normally, NPs are predominantly restricted to endocytic vesicles, thus preventing the drugs from exerting their effects. However, the ROS conversion NPs solve this problem through triggering cytosolic release of the chemotherapeutics ^104^. Wei et al.^105^ have developed photoconversion NPs that cause photochemical rupture of lysosomal membranes under 635 nm (10 mW/cm^2^, 5 min) NIR and release drugs into the cytoplasm (**Figure 5D**). The establishment of intelligent nano-systems thus can improve the low tumor specificity of PDT and SDT.

### Co-delivery of multiple therapies

The application of multi-dimensional therapy can be achieved through nano-platforms^106–108^. Single PDT or SDT cannot completely solve drug-resistance issues and usually must be supplemented with exogenous oxygen, inhibitors, targeting agents, and immunotherapy. Nanotechnology has made this combination possible.

**Figure 6:**
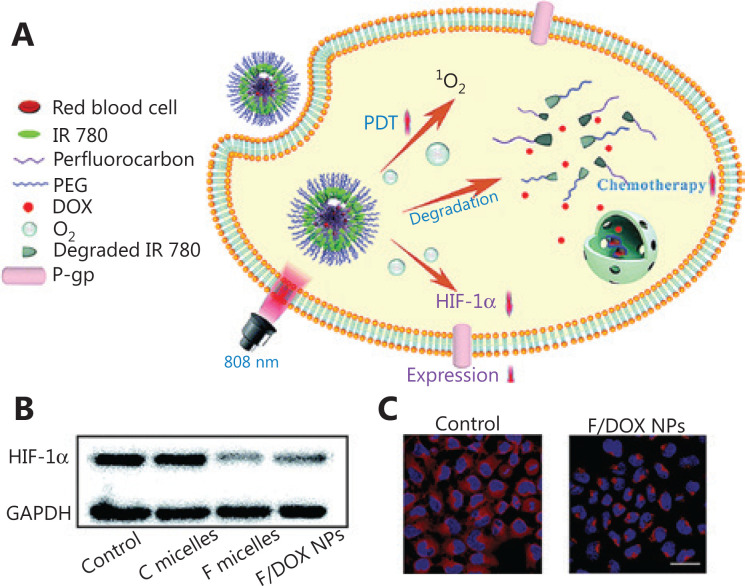
(A) A nanoparticle formulation co-delivers O2 and IR780, thus overcoming resistance of chemotherapy and PDT caused by hypoxia. (B) HIF-1 expression after treatment of MCF-7 cells with different methods. (C) CLSM images of P-gp expression after treatment with F/DOX nanoparticles under hypoxic conditions^113^. Copyright 2019, Royal Society of Chemistry.

1. The ABC protein inhibitors have a good effect on reducing drug efflux^109^. After treatment combining inhibitors with PDT and SDT, drug-resistant cancer cells are limited^110,111^. Wei et al.^105^ have used a nano-platform to combine PDT and the ABC protein inhibitor apatinib. In drug-resistant breast cancer, the IC_50_ for this combination therapy is 17.34 μg/mL, and the apoptotic ratio is 45.34%, a value approximately 3.3-fold higher than that of free DOX.
2. Hypoxia, one of the main features of TME, increases the resistance of chemotherapy, PDT, and SDT. Nanotechnology provides tools to regulate the TME and re-sensitize tumors^112^. Yang et al.^113^ have synthesized oxygen self-sufficient NPs (F/DOX) loaded with oxygen-bearing perfluorocarbon and DOX. Under 808 nm light excitation, this nanocarrier disintegrates (**Figure 6A**). The release of oxygen decreases the expression of HIF-1, and correspondingly low expression of P-gp has also been observed in CLSM (**Figure 6B–C**). McEwan et al.^114^ have developed an oxygen-containing microbubble system for the targeted treatment of pancreatic cancer. When micro-bubbles are exposed to US (1 MHz, 3.0 W/cm^2^, 50% duty cycle, 1 min), oxygen is released, thus leading to downregulation of HIF-1.
3. EMT enables cancer cells to invade and metastasize like CSCs, thus contributing to drug resistance^115^. Some NPs have been designed to target EMT cells and CSCs to hinder tumor escape and drug resistance^116,117^. Spring et al.^118^ have synthesized photoactivated nanoliposomes combined with inhibitors, integrating photodynamic and anti-VEGF therapy. This treatment causes blood vessel damage and blocks the EMT pathway. Liu et al.^119^ have constructed NPs co-loaded with HP and DOX. When combined with US radiation (1.0 MHz, 3 W/cm^2^, 5 min), this treatment effectively reverses the drug-resistance of CSCs.
4. Checkpoint-blocked immunotherapy provides a promising strategy for cancer therapy, although its effects on low T cell infiltration tumors remain limited^120^. PDT and SDT with immunogenicity induce tumor cell sensitivity to PD-L1 immunotherapy by initiating an inflammatory response^121,122^. He et al.^123^ have designed core-shell NPs carrying pyropheophorbide and oxaliplatin for enhanced immune checkpoint suppression therapy. Under light (670 nm, 60 mW/cm^2^, 15 min) irradiation, the apoptosis rate of HT29 cells is 43%, and the necrosis rate is 18.7%. These NPs cause immunogenic cell death and inflammatory responses at the primary tumor location, thus stimulating the proliferation of effector T cells and enhancing the efficacy of PD-L1.

## Summary and outlook

The emergence of drug resistance has made inhibiting the proliferation of cancer cells more difficult. PDT and SDT are widely used to combat drug-resistant tumors. First, the ROS produced by PDT and SDT destroy subcellular structural membranes, DNA, and proteins, in contrast to the traditional anti-cancer pathway. Second, ROS inhibit the expression of ABC proteins, thus directly decreasing the efflux of drugs. Finally, PDT and SDT also contribute to overcoming drug resistance by damaging the vasculature and sensitizing tumor cells. However, the low specificity and water solubility of sensitizing agents limit the clinical applications of PDT and SDT.

NPs provide powerful tools to treat drug-resistant tumors on the basis of the EPR effect, owing to their structural designability. Unlike small molecule drugs, NPs enter cells through endocytosis. The combination of smart nanomaterials with PDT and SDT improves the targeted delivery and solubility of sensitizing agents, thus maximizing the therapeutic efficacy of the combinational therapy. For instance, NIR irradiation of the sensitizer generates ROS *in situ*, thus resulting in PEG de-shielding at the tumor region and significantly enhancing the cellular uptake of the nanocarrier. Light-induced PEG shedding enables precise remote control of drug delivery by de-shielding nanocarriers. In general, the synergy maximizes the effect in overcoming drug resistance. Such nanodrug delivery systems have promising therapeutic efficacy.

Although nanotechnology greatly aids in PDT and SDT, many problems remain to be addressed. (1) The low tissue penetration of NIR and quantum yield because of aggregation remain the main reasons for the poor therapeutic effects; consequently, PS with longer excitation wavelengths and delivery strategies that can avoid aggregation must be developed. (2) Further research on cavitation and the mechanical mechanism of SDT is needed. A more comprehensive understanding of the interaction between the sensitizer and cavitation, and the design of a more appropriate delivery system to enhance cavitation, would be conducive to SDT. (3) The low loading rate, instability, and potential toxicity of nanomaterials remains to be addressed. (4) Currently, we are in the new era of immunotherapy. Antibody-based PD-1/PD-L1 blockaded therapy has achieved dramatic therapeutic responses. However, this therapy is only effective for a subset of patients. Patients who do not respond to immunotherapy are referred to “primary resistance”. PDT and SDT provided a powerful toolbox to resolve this issue. Hence, more comprehensive studies related to immunotherapy and PDT/SDT are highly desired. (5) Although a plethora of studies have used nanotechnology to facilitate PDT and SDT and avoid drug resistance, these studies have mainly focused on the inhibitory effects of drug-resistant cancer cells and the synergistic effect of PDT/SDT and chemotherapy. The intracellular mechanism of this therapy in overcoming drug resistance remains unclear. Notably, nanotechnology-assisted PDT and SDT should be advantageous in overcoming long-lasting challenges in drug resistance, although this combination therapy remains in early stages. The comprehensive development of better nano-delivery systems and high-efficiency sensitizers are future directions toward achieving applications in clinical treatment.
